# Novel phenotype characterization utilizing electrical impedance myography signatures in murine spinal cord injury neurogenic bladder models

**DOI:** 10.1038/s41598-023-46740-6

**Published:** 2023-11-09

**Authors:** Hsin-Hsiao Scott Wang, Hatim Thaker, Alex Bigger-Allen, Janice A. Nagy, Seward B. Rutkove

**Affiliations:** 1 Department of Urology, Boston Children’s Hospital, 300 Longwood Ave, HU390, Boston, MA USA; 2 Harvard Medical School, Boston, MA 02215 USA; 3 Department of Neurology, Beth Israel Deaconess Medical Center, Boston, MA USA

**Keywords:** Urology, Biophysics

## Abstract

Neurogenic bladder (NB) affects people of all ages. Electric impedance myography (EIM) assesses localized muscle abnormalities. Here, we sought to investigate whether unique detrusor EIM signatures are present in NB due to spinal cord injury (SCI). Twenty-eight, 8–10 weeks old, C57BL/6J female mice were studied. Twenty underwent spinal cord transection; 8 served as controls. Cohorts were euthanized at 4 and 6 weeks after spinal cord transection. Each bladder was measured in-situ with EIM with applied frequencies of 1 kHz to 10 MHz, and then processed for molecular and histologic study. SCI mice had greater bladder-to-body weight ratio (*p* < 0.0001), greater collagen deposition (*p* = 0.009), and greater smooth-muscle-myosin-heavy-chain isoform A/B ratio (*p* < 0.0001). Compared with the control group, the SCI group was associated with lower phase, reactance, and resistance values (*p* < 0.01). Significant correlations (*p* < 0.001) between bladder-to-body weight ratios and EIM measurements were observed across the entire frequency spectrum. A severely hypertrophied phenotype was characterized by even greater bladder-to-body weight ratios and more depressed EIM values. Our study demonstrated distinct EIM alterations in the detrusor muscle of mice with NB due to SCI. With further refinement, EIM may offer a potential point-of-care tool for the assessment of NB and its response to treatment.

## Introduction

Neurogenic bladder (NB) is a condition that widely affects people of all ages. It results in urinary incontinence, lower urinary tract symptoms such as urinary frequency, urgency, and pain, urinary retention, and urinary tract infection. NB is fundamentally caused by aberrant or interrupted neural control of the bladder and is commonly seen in patients with a wide range of conditions, including, diabetes, pelvic surgery such as prostatectomies, and those with primary neurologic conditions such as, multiple sclerosis, spinal cord injury, and spina bifida^1–3^. Not surprisingly, NB is associated with a significant healthcare burden with billions of dollars spent annually in the United States alone^4^. The urodynamic study (UDS) is considered the gold standard to evaluate and monitor bladder function in these patients. However, the interpretation is challenging and is associated with significant inter-observer variability due to arbitrarily defined metrics^5, 6^. Therefore, there is a gap in our ability to generate objective, quantifiable, and reproducible UDS parameters, which can lead to variable and inadequate clinical care in NB patients.

Electric impedance myography (EIM), essentially a form of localized bioimpedance measurement, is a technique that has been developed for the evaluation of neuromuscular disorders. Its applications include use in neuromuscular diagnosis and monitoring of disease progression such as in amyotrophic lateral sclerosis (ALS) and Duchenne muscular dystrophy (DMD)^7–10^. It was found that alterations in skeletal muscle structure and composition such as myocyte hypertrophy and atrophy, inflammation, edema, and connective tissue and fat deposition impact the measured impedance^11, 12^. Some of these histological features are also shared in bladder structural changes in NB^13, 14^. In addition, EIM has many attractive advantages in clinical care, including high sensitivity to structural and biophysical properties, numerical data outputs that do not require complex image analysis or detailed operator interpretation, relatively high reproducibility, and its being easy to perform non- or minimally- invasively^15^. Given its overall simplicity of use and its requirement of only having four electrodes in the proximity of muscle tissue, EIM could theoretically also be applied readily to assess bladder wall integrity and health. Various structural changes on imaging, and histology have been described in NB patients and include detrusor fibrosis, edema, inflammatory infiltration, dilation, and smooth muscle hypertrophy^13, 14, 16^. Given these changes, EIM could provide a unique opportunity to study NB-associated biophysiological and structural alterations in a non-invasive, real-time, yet objective and reproducible fashion.

In spinal cord injury (SCI), the micturition reflex is eliminated in the subacute phase with storage dysfunction developing over time (e.g. detrusor overactivity, bladder hypertonicity) along with voiding dysfunction (e.g. detrusor sphincter dyssynergia)^17^. These changes induce bladder remodeling in a three-phase manner, including hypertrophy, compensation, and decompensation^18^. In the initial phase, smooth muscle cell hypertrophy within the detrusor is a hallmark of the low flow/high pressure system seen in outlet obstruction. In addition, extracellular matrix remodeling contributes to inter- and intra-fascicular collagen and elastic fiber deposition. The murine spinal cord injury (SCI) model mimics these changes and therefore is commonly utilized to study NB. The SCI induced bladder tissue changes are thought to be critical to bladder functional deteriorations but difficult to characterize with current clinical diagnostic tests. This gap in clinical care can theoretically be filled by utilizing the unique aspects of EIM with its superior ability to monitor compositional changes in muscle tissue. In addition, SCI model has the added benefit of wide transgenic manipulations and therefore evaluation of specific pathways; it is also much more cost-effective than larger animals.

In this study, we sought to investigate the feasibility of using EIM to detect distinct changes in the detrusor muscle in a murine SCI model. Based on the ability of EIM to detect and quantify changes in skeletal muscle structural composition in subjects affected by ALS or DMD, such as fibrosis, edema, cellular hypertrophy and/or atrophy, we hypothesized that EIM may also be sensitive to changes in the biophysical and structural alterations of the bladder detrusor muscle in NB and that these alterations would correspond to distinct histological and molecular features of the tissue.

## Materials and methods

### Animals

All animal studies were conducted at Boston Children’s Hospital and approved under Institutional Animal Care and Use Committee (protocol number 19-12-4065). Studies were performed in accordance with the guidelines of the Association for Assessment and Accreditation of Laboratory Animal Care International. Boston Children’s Hospital institutional animal care program holds an active animal welfare assurance on file with the Office of Laboratory Animal Welfare at the National institutes of Health, which describes the hospital adherence to Public Health Service’s Policy on Humane Care and Use of Laboratory Animals. This study was also performed in accordance with the ARRIVE guidelines^19^. Female C57BL/6J mice (Charles River), were used age 8–10 weeks, weight 18–22 g). All mice were housed with five mice per cage, with free access to water and pelleted diet ad libitum.

### Spinal cord injury (SCI) murine model

Female mice were anesthetized using 2–3% isoflurane, and a 1 cm dorsal incision was made to expose the thoracic spinal column. Through a mid-thoracic (T8-T10) laminectomy, the spinal cord was completed transected (to our knowledge to be one of the most reproducible ways of producing a consistent phenotype, which for bladder-related study, includes a phenotype of detrusor-sphincter dyssynergia, neurogenic detrusor overactivity as well as bladder tissue fibrosis). The incision was closed with absorbable suture (5–0 Vicryl, Ethicon). Post-operative care included analgesia with sustained-release buprenorphine (1 mg/kg), and 0.9% normal saline with enrofloxacin injections for three days to prevent UTI.

To assess the completeness of spinal cord transection, we confirmed that all mice had bilateral hindlimb paralysis immediately after surgical spinal cord transection. The animals moved using only forelimb activity and dragged their hind limbs. These mice were in spinal shock, resulting in urinary retention that required manual bladder expression to evacuate urine every 12 h for up to 14 days post-operatively. Activity and alertness levels otherwise remained the same as uninjured mice. Loss of proprioception or sensation was not directly assessed and recorded but assumed to be present given the complete transection. There was no variability in the technique of spinal cord transection or post-operative care that we believe could have resulted in a difference in phenotype. Two groups (SCI and Wild-Type non-instrumented control animals with normal bladders) were studied^20^.

At 4 or 6 weeks after SCI, depending on cohort (described below), individual mice were placed in a rectangular polycarbonate cage without wire flooring, lined with Whatman paper on the bottom (Cat #3030-917), from 8 pm to 12 am. Mice were fed ad libitum, but water was restricted to avoid water dripping onto the filter paper. Papers were dried and imaged via ChemiDoc MP imaging system (UV trans illumination, 0.7 s exposure time) to visualize voided spots of urine (voiding spot assay).

A first cohort (3 control, 10 SCI) was followed and euthanized 4 weeks after SCI. A second cohort (4 control, 9 SCI) was euthanized 6 weeks after SCI to observe potential longer-term structural dysfunction impacted by SCI.

### Electrical impedance myography

**Figure 1:**
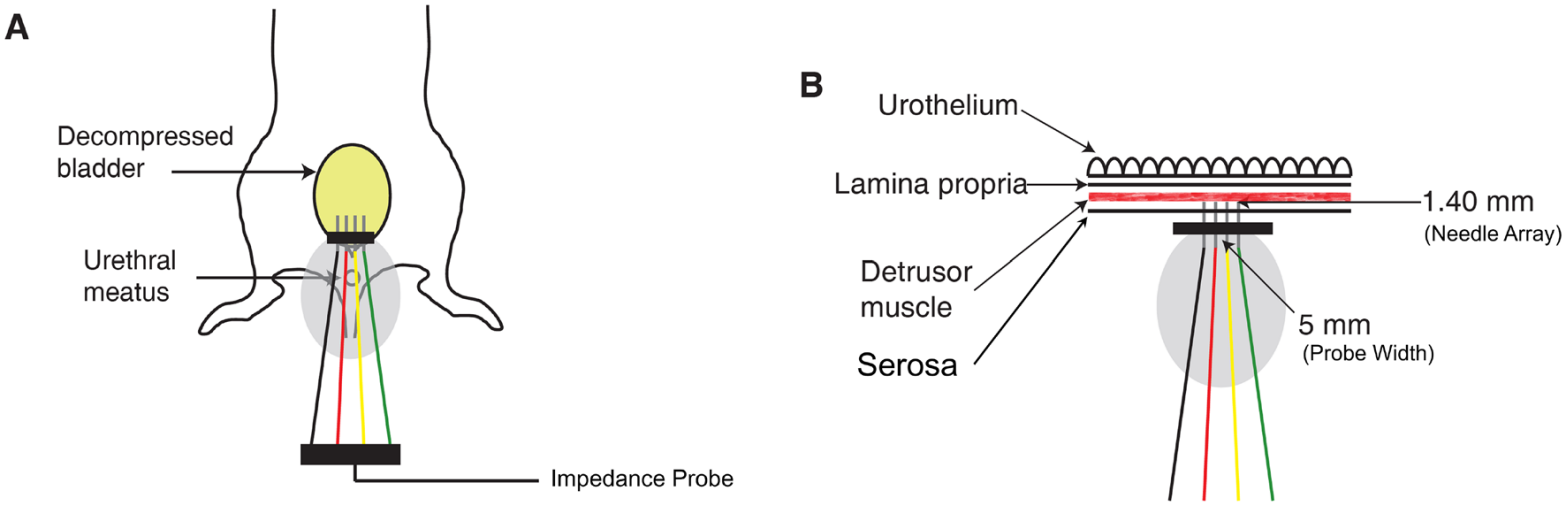
Electrical Impedance Myography Set-up for murine bladder detrusor muscle. (**A**) EIM measurement in freshly post-mortem mice. The impedance probe was placed gently on decompressed murine bladder. (**B**) Magnified view of the spatial relationship between EIM probe and bladder detrusor muscle.

Mice were euthanized via CO_2_, and immediately underwent a low midline laparotomy to expose the bladder. The bladder was then manually compressed to evacuate all residual urine. Post-mortem impedance measurements of the bladder were performed in situ using the mView System (Myolex, Inc, Boston, MA), with 41 frequencies ranging from 1 kHz to 10 MHz. Measurements were made using a fixed 5 mm wide needle array (1.4 mm deep)^21^ that was positioned on the serosal surface to superficially puncture the detrusor muscle. The EIM measurement technique is illustrated in Fig. 1.

The three major impedance variables measured include the resistance (measurement of difficulty passing current through the tissue), the reactance (measurement of the capacitive effects of the cell membranes), and the phase (a trigonometric proportion of these two values, obtained via the equation, phase = arctan (reactance/resistance)). Given artifacts that occur at the extremes of the frequency range, frequencies between 1 kHz and 1 MHz were used for all analyses that follow.

Following EIM data collection, the bladders were harvested, weighed, and allowed to equilibrate in Hank’s buffered saline solution for 5 min. Hemi-bladders then underwent formalin fixation or were flash frozen in liquid nitrogen.

### Histology

Bladder tissues were fixed in 10% formalin overnight, embedded in paraffin, and sectioned (8 µm thick). Sections were subjected to Masson’s Trichrome staining and digitally imaged via an Olympus DP73 color camera. A macro was written to quantify collagen intensity in the detrusor smooth muscle of bladder sections on the digital images. In brief, the macro performs 3 steps: (1) raw RGB images are duplicated and a threshold is applied to generate a region of interest (ROI) identifying the entire tissue in the image, (2) a second copy of the raw RGB image is separated into three colors corresponding to the pigments used to stain the slides using the color deconvolution plugin (FIJI/ImageJ, National Institutes of Health) and the image containing the red pigment corresponding to the plasma stain was used to generate the ROIs that identify the detrusor smooth muscle, and (3) the ROIs were superimposed onto the image corresponding to the fiber stain identifying collagen and the integrated density was measured for each. The integrated density of collagen signal in the detrusor was normalized to the detrusor area as a fraction of the total tissue area.

### RNA isolation and RT-qPCR

Following tissue homogenization with Fastprep-24 (MP Biomedicals), RNA was isolated with Trizol and RNAeasy Mini Kit (Qiagen), per manufacturer’s protocol. RNA was quantified on a Nanodrop-2000 instrument, ensuring a minimum of 100 ng/μl were purified, and equal amounts (1 μg of RNA) of each sample were reverse transcribed into cDNA with SuperScript III RT Kit (Invitrogen) with oligo-dT primers.

Levels of smooth muscle myosin heavy chain isoform (SM-A without head insert and SM-B with head insert) were assayed by semi-quantitative reverse transcription polymerase chain reaction (RT-PCR) using gene-specific primers (SM-A/SM-B, Forward (5′-CCA CAA GGG CAA GAA AGA CAG C-3′) and Reverse (5′-TCC GGC GAG CAG GTA GTA GAA GA-3′), Integrated DNA Technologies), and run on 3% agarose gel. Relative expression levels were quantified via the Gel Analyzer plugin in FIJI.

### Statistical analysis

Bivariate analyses were performed to compare SCI versus healthy controls. Multifrequency analysis was performed and calculated from the EIM values obtained between 1 kHz and 1 MHz. We used the t-test, or Wilcoxon rank-sum test, as appropriate, based on data characteristics and distribution (such as normality using Shapiro–Wilk test). ANOVA was used to compare the EIM measurements (phase, reactance, resistance) in three groups (control, SCI/dilated phenotype, SCI/hypertrophied phenotype), followed by group-wise multiple group comparison with Dunnett’s test for repeated measurements. Pearson’s correlation coefficient was calculated to assess the relationship between the bladder-to-body weight ratio, or the amount of collagen deposition, and various EIM measurements at selected frequencies. Due to the limited bladder tissue, we were able to conduct MTS staining and SM-A/SM-B mRNA analysis only in 13 subjects (3 controls, 10 SCI, no hypertrophy phenotype) in the 4-week cohort. An alpha of 0.05 (two-tailed) and 95% confidence intervals (CI) were used as criteria for significance. All analyses were performed using R 0.4.0 and GraphPad Prism 9.1.0.

## Results

### Basic functional comparison of SCI and control mice

**Figure 2:**
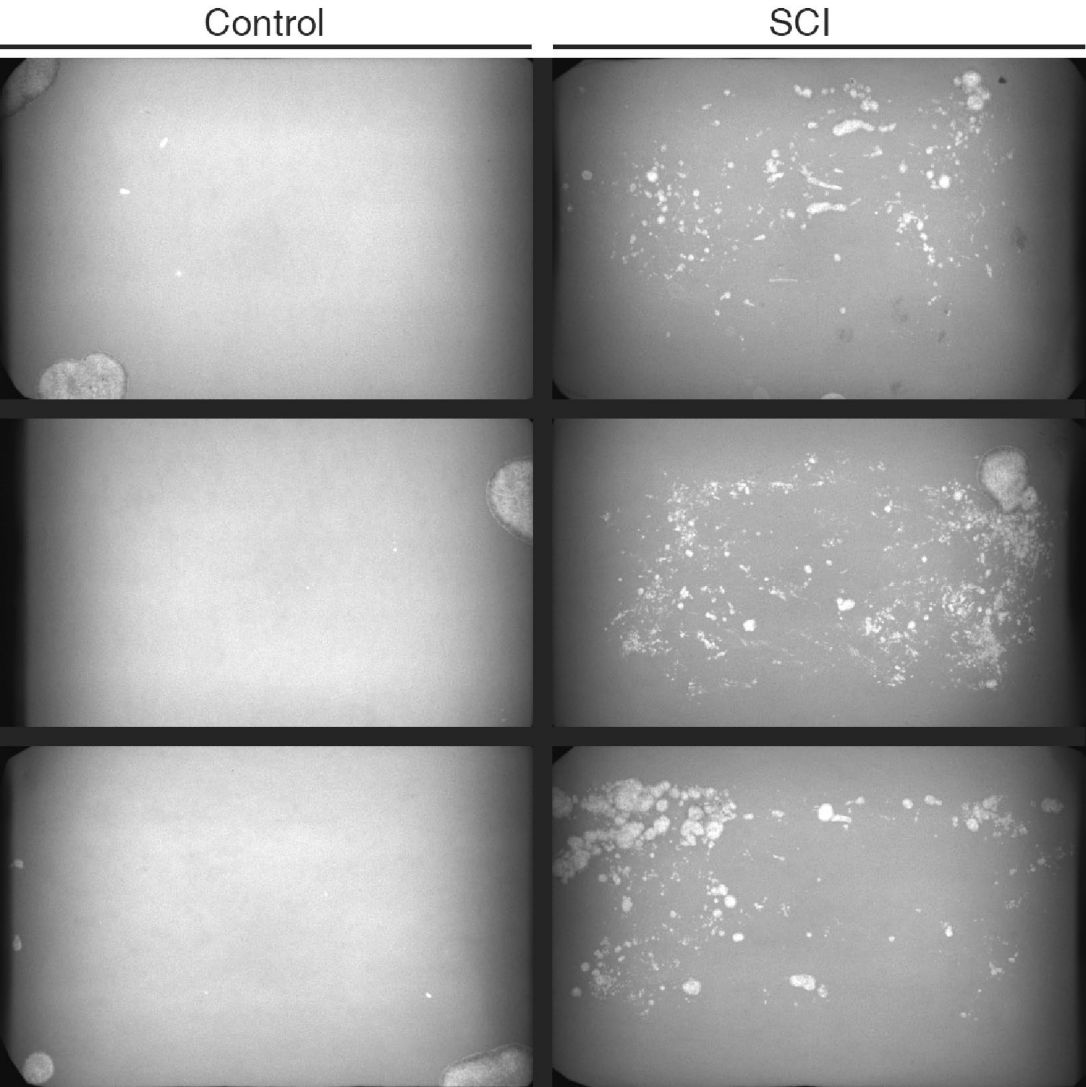
Functional analysis using the Voiding spot assay in Control and SCI mice. Control mice (left) demonstrated few voiding spots indicating volitional and controlled voiding. The SCI mice (right) showed constant dribbling of urine across the cage indicating uncontrolled voiding.

The Voiding spot assay, conducted 4 weeks post spinal cord transection, showed successful SCI surgery and subsequent bladder dysfunction with constant dribbling of urine across the cage for mice from the SCI group, whereas mice from the control group demonstrated few voiding spots indicating volitional and controlled voiding (Fig. 2). Confident that the SCI surgery resulted in the expected changes in micturition pattern, we proceeded with further bladder analysis.

### Physiological, histological analysis of control and SCI bladders

**Figure 3:**
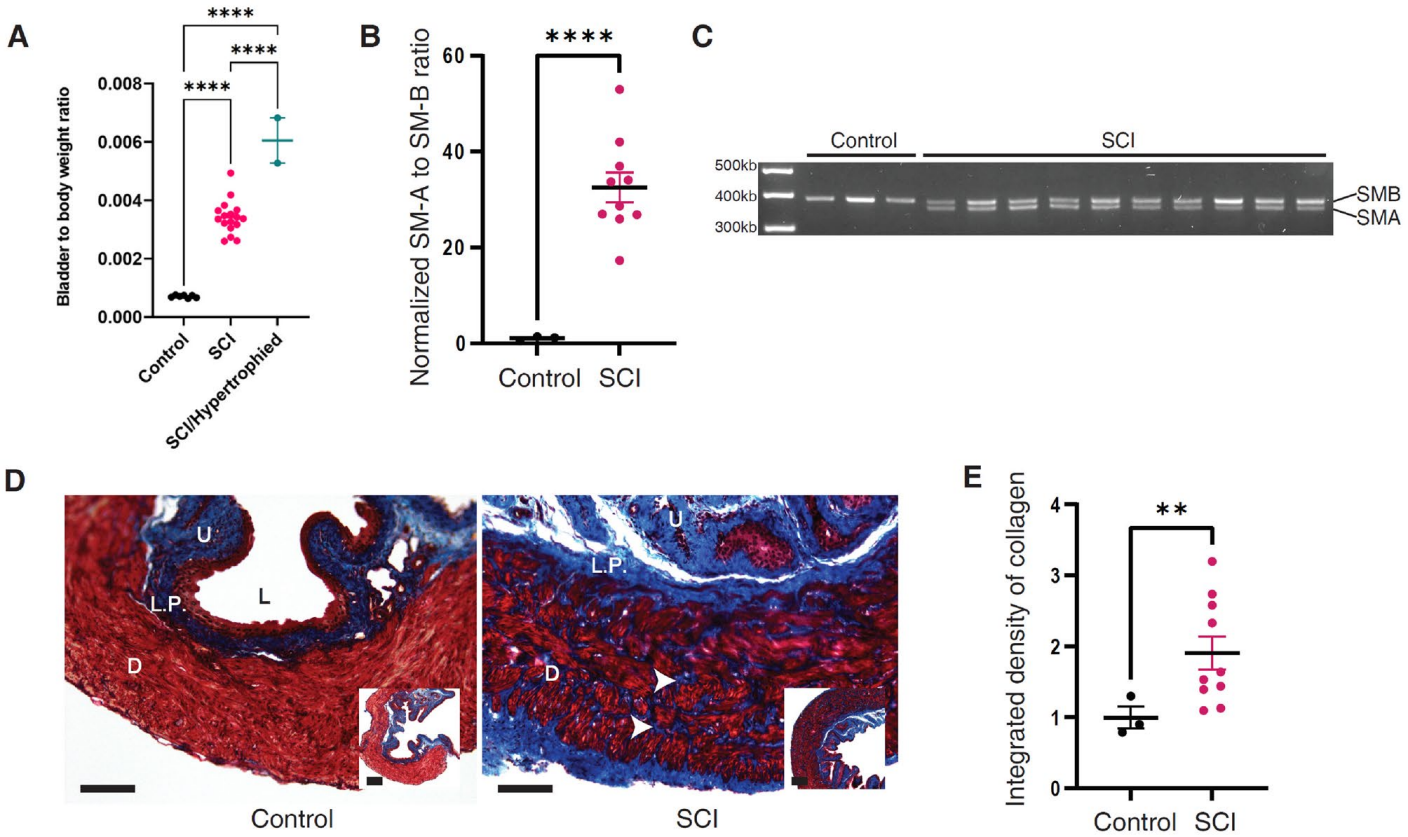
Physiological, histological, and molecular analysis of Control and SCI Bladders. (**A**) Bladder-to-Body weight ratio comparison between control, SCI, and SCI with severe hypertrophy groups. (**B**) SM-A to SM-B ratio comparison between SCI and control bladders. The comparison was performed with 13 subjects (3 Control and 10 SCI at 4-week group). (**C**) RT-PCR analysis showing both SM-A and SM-B expression in SCI bladders. Control bladders express only SM-B; SCI bladders express both SM-A and SM-B. The comparison was performed with 13 subjects (3 Control and 10 SCI at 4-week group). The Full length gel is reported in the supplementary information section. (**D**) Histological Analysis (MTS staining) comparison between control and SCI bladders. Note that bladder detrusor muscle (red staining) is thinner and homogeneous in the Control sample. Much more collagen deposition (blue staining) is visible in the detrusor muscle in the SCI sample. D- detrusor, L.P.- Lamina propria, U- Urothelium, L- Lumen, Scale Bar = 100 μm in both enlarged and small insets. The comparison was performed with 13 subjects (3 Control and 10 SCI at 4-week group). (**E**) Collagen density (detrusor area/total area) comparison between SCI and control (control as reference set to equal 1, analysis performed in 3 Control and 10 SCI at 4-week group). In Panels (**A**), (**B**) and (**E**): *: *p* < 0.05, **: *p* < 0.01, ***: *p* < 0.001, ****: *p* < 0.0001.

By convention, and consistent with previous studies reporting outcomes of NB secondary to rodent SCI, absolute bladder wall thickness is not reported since there is variability in where measurements are taken (the bladder dome thickness will be different than the bladder base). To avoid this inaccuracy, whole bladder to body weight ratios are reported instead. The SCI mice had significantly higher bladder weight while the body weight was similar to control (bladder-to-body weight ratio mean at [3.4 ± 0.6] × 10^–3^ in SCI vs [7.0 ± 0.5] × 10^–4^ in control, Fig. 3A). Two of the 9 SCI mice developed a bladder hypertrophy phenotype with severely thickened bladder wall and bladder stone formation. The bladder-to-body weight ratio was almost twice as large for the SCI/hypertrophied group compared with SCI group with the mean at (6.0 ± 1.1) × 10^–3^.

As depicted histologically in Fig. 3D, the SCI bladder detrusor layer appears thickened with significant collagen deposition (blue infiltration) compared to the uniform appearance of the detrusor smooth muscle layer (homogeneous red) in the control bladder. Compared to control, SCI bladder tissue was associated with significantly higher density of collagen in the detrusor layer (1.9 times more comparing to control, *p* = 0.009) (Fig. 3E).

### Molecular analysis of control and SCI bladders

In terms of smooth muscle structural mRNA expression variations, bladder tissue from the control group expressed only the SM-B smooth muscle myosin heavy chain isoform, which indicates normal detrusor smooth muscle. On the other hand, bladder tissue from the SCI group expressed both SM-A and SM-B isoforms (Fig. 3C) indicating smooth muscle tissue remodeling with a much higher normalized SM-A to SM-B ratio (32.5 in SCI vs. 1 as control, *p* < 0.0001) as shown in Fig. 3B.

### Electrical impedance myography measurements and analysis

Compared with the control group, the SCI group had significantly lower EIM values (phase, reactance, and resistance). This difference was even more prominent in the two SCI mice from the 6-week group demonstrating significant detrusor hypertrophy. The EIM values obtained in the hypertrophy group were significantly lower than the values obtained for the other SCI mice for all 3 impedance measures across the entire spectrum. Therefore, the EIM values were observed to be consistently as follows: SCI/hypertrophied < SCI < controls.

**Figure 4:**
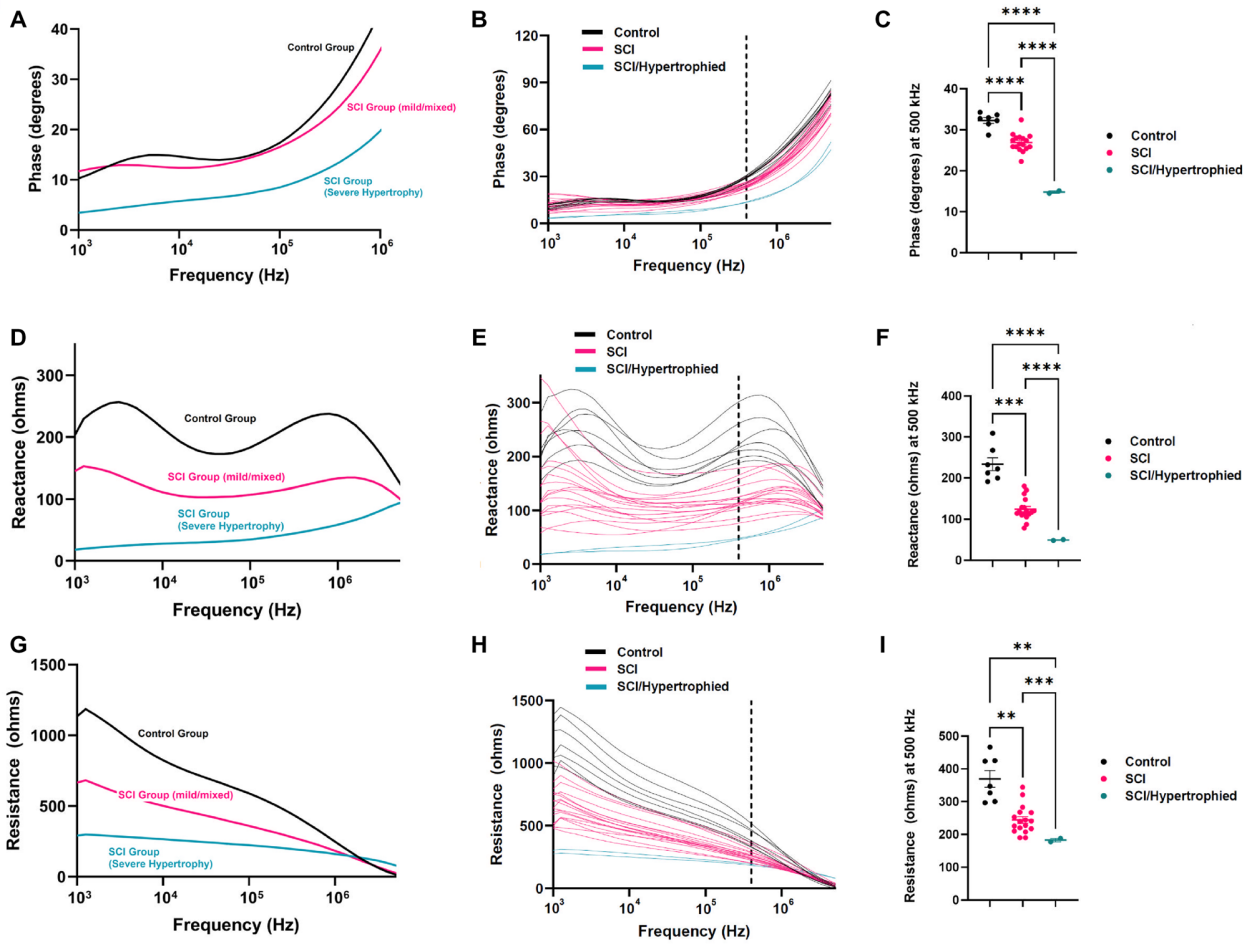
EIM measurements for Control, SCI/Mild and SCI/Hypertrophied Bladders. (**A**, **B**, **C**) Phase values for Control and SCI groups. (**A**, **B**) Multifrequency plots of phase values for control, SCI with mild NB changes, SCI with severe hypertrophy. Whereas the control and the mild SCI groups had similar phase values, the SCI with severe hypertrophy group had distinctly lower phase values over the entire frequency range. Significant separation among the three groups became prominent at frequencies between 10^5^ and 10^6^ Hz. (**C**) 6-week SCI/hypertrophied group showed a significantly reduced mean phase value compared to both the control and the other SCI group at 500 kHz. *: *p* < 0.05, **: *p* < 0.01, ***: *p* < 0.001, ****: *p* < 0.0001. (**D**, **E**, **F**) Reactance values for Control and SCI groups. (**D**, **E**) Multifrequency plots of reactance values for control, SCI with mild NB changes, SCI with severe hypertrophy phenotypes. The three groups had distinctly different reactance profiles over the entire frequency range. (**F**) 6-week hypertrophied group showed a significantly reduced mean reactance value compared to both the control and the other SCI group at 500 kHz. *: *p* < 0.05, **: *p* < 0.01, ***: *p* < 0.001, ****: *p* < 0.0001. (**G**, **H**, **I**) Resistance values for Control and SCI groups. (**G**, **H**) Multifrequency plots of resistance values for control, SCI with mild NB changes, SCI with severe hypertrophy. These three groups had distinctly different resistance profiles over the entire frequency range. (**I**) 6-week SCI/hypertrophied group showed a significantly reduced mean resistance value compared to both the control and the other SCI group at 500 kHz. *: *p* < 0.05, **: *p* < 0.01, ***: *p* < 0.001, ****: *p* < 0.0001.

The full multifrequency EIM spectra for all animals (combining both the 4- and 6-week groups and separating the SCI animals into 2 groups, i.e., hypertrophied versus non-hypertrophied) are shown in Fig. 4. Figure 4A–C show phase values, Fig. 4D–F show reactance values, and Fig. 4G–I show resistance values. For both reactance and resistance measurements, the values for the control group were higher across most of the frequency spectrum. For phase measurements, the clearest separation between the control and the SCI groups was observed between 10^5^–10^6^ Hz. The consistent largest visual separation among all three groups for all three parameters was observed at a frequency of 500 kHz (marked by vertical dashed lines in the multifrequency EIM plots in Fig. 4B, E, and H). The corresponding mean impedance values at this frequency are shown in the scatter plots in Fig. 4C, F, and I.

At 500 kHz, the impedance results included: lower phase values (mean SCI /hypertrophied 15 ± 0.4° vs. SCI 26 ± 2° vs. control 32 ± 2°, *p* < 0.0001), lower reactance values (mean SCI/hypertrophied 50 ± 1Ω vs. SCI 125 ± 27Ω vs. control 233 ± 42Ω, *p* < 0.0001), and lower resistance values (mean SCI/hypertrophied 183 ± 7Ω vs. SCI 244 ± 42Ω vs. control 370 ± 68Ω, *p* < 0.0001). A comparison of EIM values for the control, SCI and SCI/hypertrophied groups at 5, 50, 100 and 500 kHz, along with corresponding p-values, can be found in Table 1.

**Table 1:** Comparison of mean EIM values at different frequencies.

	Frequency (kHz)	Control (N = 7)	SCI (N = 17)	SCI-hypertrophied (N = 2)	ANOVA *p* value
Phase (°)	5	15	13	5	< 0.0001
	50	15	14	7	< 0.0001
	100	17	17	8	< 0.0001
	500	32	27	15	< 0.0001
Reactance (Ω)	5	247	126	26	< 0.0001
	50	173	104	32	< 0.0001
	100	183	107	35	< 0.0001
	500	233	125	50	< 0.0001
Resistance (Ω)	5	934	550	277	< 0.0001
	50	656	403	236	< 0.0001
	100	589	359	223	< 0.0001
	500	370	244	183	< 0.0001

### Correlation between EIM values and physiologic and histologic markers

As shown in Table 2, higher bladder-to-body weight ratio is significantly correlated with lower phase, reactance, and resistance values across the frequency spectrum.

**Table 2:** Correlation between Bladder-to-Body weight ratio and EIM measurements.

Frequency (kHz)	Correlation coefficient with Phase	*p* value
5	− 0.69	< 0.0001
50	− 0.52	0.006
100	− 0.57	0.002
500	− 0.81	< 0.0001

Frequency (kHz)	Correlation coefficient with Reactance	*p* value
5	− 0.73	< 0.0001
50	− 0.68	0.0001
100	− 0.70	< 0.0001
500	− 0.72	< 0.0001

Frequency (kHz)	Correlation coefficient with Resistance	*p* value
5	− 0.79	< 0.0001
50	− 0.78	< 0.0001
100	− 0.77	< 0.0001
500	− 0.70	< 0.0001

*Bladder-to-body weight correlation was performed with N subjects (7 Control and 17 SCI and 2 SCI/hypertrophied).

We also found increased collagen deposition (using MTS staining) was correlated with lower phase and reactance values as shown in Table 3. Additionally, we found stronger but non-significant correlations between collagen deposition and EIM measurements at low frequency.

**Table 3:** Correlation between Collagen deposition (MTS assay) and EIM measurements.

Frequency (kHz)	Correlation coefficient with Phase	*p* value
5	− 0.54	0.06
50	− 0.23	0.45
100	− 0.35	0.24
500	− 0.45	0.13

Frequency (kHz)	Correlation coefficient with Reactance	*p* value
5	− 0.43	0.13
50	− 0.25	0.41
100	− 0.27	0.37
500	− 0.25	0.40

Frequency (kHz)	Correlation coefficient with Resistance	*p* value
5	− 0.21	0.49
50	− 0.18	0.55
100	− 0.18	0.57
500	− 0.03	0.92

*MTS (staining for collagen deposition) correlation was performed with 13 subjects (3 Control and 10 SCI).

## Discussion

Our SCI animal model resulted in NB phenotypes in the SCI group with evidence of an increased ratio of bladder to whole body weight and constant dribbling voiding pattern in voiding spot assays. Expected structural variations were also clearly noted histologically and molecularly with increased collagen deposition observed in MTS staining and specific mRNA expression (increased SM-A in comparison to routine SM-B expressions^22^) indicating bladder fibrosis, inflammatory and remodeling changes in NB of the SCI group, consistent with previous reports^23, 24^.

Multi-frequency EIM can provide insights into both extracellular (observed at lower frequency) and intracellular (observed at higher frequency) changes in the tissue of interest^15^. In this study, we found that the EIM signature has the potential of identifying bladder detrusor changes resulting from SCI, with distinct differences in all three EIM parameters. Compared to control bladders, SCI mouse bladder from both the mild and hypertrophied groups had distinct EIM signatures across the frequency spectrum. The reason for this is likely related to the major alterations observed on histological examination. NB changes include a combination of substantial collagen tissue deposition, tissue edema, and inflammation. Taken together, these changes would lead directly to lower resistance values especially at low frequency since these changes are present predominantly in the extracellular space. Edema, due to an increase in free water, will lower resistance but so will fibrosis due to connective tissue deposition, which has relatively low resistivity values compared to healthy muscle tissue^25^. Additionally, previously reported increased gap junction expression and coupling in SCI and obstructed bladder could also lead to lower resistance^26, 27^. The stronger (but non-significant) correlations between the degree of collagen deposition on histology mainly at low frequencies supports this. Moreover, the lower reactance values observed across the entire frequency spectrum are also consistent with reduced myofiber size (associated with reduced lipid containing myofiber membrane material) and increased less “chargeable” materials (e.g., collagen and edema). This also directly impacts the spectral characteristics, resulting in a diminution of the peaks normally seen in the reactance spectra (see Fig. 4, Panel D). To some extent, these changes are counterbalanced in the calculation of the phase (which is a ratio between the reactance and resistance values) and so alterations are less apparent, except in the most severe animals, where the profound changes in reactance outweigh those observed in the resistance. Furthermore, the significant correlation between the higher bladder-to-body weight ratio and lower EIM measurements provide further support this hypothesis.

This is one of the first studies investigating in situ EIM in smooth (detrusor) muscle as opposed to the significant body of work previously reported using EIM to evaluate skeletal muscle in both pre-clinical and clinical settings. Interestingly, findings such as EIM measurements in pathologic detrusor muscle in SCI model are similar to EIM results obtained in patients with skeletal muscle pathology such as Duchenne muscular dystrophy^7^. More specifically, increasing connective tissue and associated detrusor loss and atrophy likely cause a shift downward in the frequency spectrum in reactance and resistance, with increasing values at higher frequencies and reductions at lower frequencies for phase value. Additionally, the SCI subtype with extreme detrusor hypertrophy was found to have even more prominent impedance changes compared to control animals and to the milder SCI phenotype (as shown in Fig. 4). To some extent, the data reported in Fig. 4 are very similar to the EIM findings in Duchenne muscular dystrophy patients demonstrating distinct EIM readings according to the severity of the disease^7^. While these are two very different disorders, we hypothesize that the histologic changes, (fibrosis, edema, and myofiber atrophy) detected in our SCI mouse bladder were similar to changes observed in Duchenne muscular dystrophy skeletal muscles (increased fat and collagen deposition) resulting in lower phase and reactance measurements.

We believe that, taken together, these data provide early support for the use of EIM to serve as a biomarker in SCI and NB research. This is especially encouraging since the current tools for phenotyping NB are very limited. At present, urologists depend largely on subjective patient symptoms and urodynamic studies to construct diagnostic and management plans that often lead to large practice variation due to the uncertain nature of patient-reported symptoms and variable urodynamic study interpretations. On the other hand, EIM provides a real-time, reliable, repeatable, objective option. Related impedance measurement approaches have been used previously for bladder assessment, but only for cancer diagnostics or estimating bladder volume estimation^28, 29^. The ability to use very small electrode arrays, as was done here, offers the possibility of studying mice and, with it, the enormous flexibility for phenotypic and genetic variability at reasonable cost.

We chose the murine SCI model to study EIM for multiple reasons. The authors have extensive experience and familiarity with the surgical creation of SCI in mice and rats, which results in a consistent NB phenotype. In this exploratory study, where we apply EIM for the first time in smooth muscle bladder tissues, we opted to use a murine model to not only reduce cost, but also because the murine model closely reflects the human SCI condition. Future studies that expand on the use of EIM in detecting bladder pathology may utilize transgenic mice to enrich our understanding of NB pathways.

The findings of our study should be viewed in the context of several design limitations. The power of the current study is limited by the small cohort and sample size and our using only female animals, which did not allow us to investigate the contribution of androgen receptor pathways in bladder remodeling and outlet obstruction^30^. The small sample size also did not allow enough statistical power to correlate the observed EIM measurement changes with alterations in phenotypical features such as increased collagen deposition. Unfortunately, due to the small amount of bladder tissue and technical challenges, we lost the majority of the tissue for the 6-week group, which limited the MTS staining and molecular analysis statistical power. Additionally, because NB in humans can have a variety of causes, this SCI model represents only a small subset of NB cases. Humans rarely have complete spinal cord transaction as a mechanism of injury and suffer mainly from partial crush injuries or acquired neural injury (e.g. spondylotic myelopathy, neoplastic compression, or vascular disease), as a cause of NB. In this exploratory study, we opted to use a complete spinal cord transection model to produce the most reliable phenotype in every animal. Yet, over time, the character of the NB alteration varied across animals, consistent with human clinical experience. Similar to other NB models such as the partial outlet obstruction model^16^, the severe hypertrophied phenotype cannot be reliably created but manifests randomly among the SCI group, thus leading to our having only 2 animals in this group. In future studies, we may assess the sensitivity of EIM to changes produced by alternate methods of SCI, such as graded crush injury (which would theoretically produce a variation in bladder phenotype) or ischemia. Nevertheless, the group studied here has significant clinical value since the pathology is similar to high intravesical pressure induced NB, characterized by infection and stone formation.

Another limitation is that our study used control mice instead of animals with sham surgery. In this early phase exploratory study, we opted to use age-matched and sex-matched control mice instead of sham mice (i.e., skin incision and laminectomy only), as previous studies have shown no difference between sham and control mice. Specifically, the study by Seth et al.^20^ demonstrated no difference in control or sham animals on histology or bladder-to-body weight ratio. The study also showed that sham animals did not display features of NB, such as detrusor overactivity. Therefore, we opted to simplify the experimental design and cost by using wild type, non-instrumented, female mice as controls. Finally, this study was conducted in situ on freshly euthanized mice to obtain clean initial exploratory data with the capability to modify parameters for physiologic evaluations. While we cannot predict our findings in live animal bladders specifically, we expect that EIM can be performed in a minimally invasive fashion in larger animals’ bladder detrusor muscle in the future based on past experience and probe design in assessing skeletal muscle in live animals. EIM has been performed with a variety of probe designs and size, and we anticipate monitoring EIM to monitor detrusor health to in vivo. For example, an EIM probe can be designed to fit the cystoscope channel to provide EIM measurement at the same time as visual assessment of the bladder. Similarly, tailored EIM leads can coat the surface of UDS catheter to provide simultaneous EIM measurement with cystometrogram and/or electromyography studies.

Despite its limitations, our study offers promise for the use of EIM to detect structural and compositional changes in the NB. This study is one of the first to demonstrate in situ EIM to objectively characterize smooth muscle (detrusor) tissue-level NB changes in the SCI mouse model. Existing diagnostic tools for assessing SCI and NB such as UDS and ultrasound provide limited information on actual bladder tissue damage. In contrast, EIM technology appears especially promising with its objective nature and potential as a biomarker for tissue-level disease monitoring and characterization for NB research and patient care. Towards this end, additional studies testing EIM’s capabilities in other animal models of NB are now being pursued.

## Conclusions

This study shows the feasibility and promise of the application of EIM to NB bladder assessment. EIM has major potential in the future phenotyping and treatment response monitoring NB patients given its real-time nature. Future studies applying EIM for bladder assessment via catheterization are now being planned.

### Supplementary Information

Supplementary Information.

## Data Availability

The datasets generated in the current study are available from the corresponding author on reasonable request.
