# Supplementary material for: Novel phenotype characterization utilizing electrical impedance myography signatures in murine spinal cord injury neurogenic bladder models
Source: Sci Rep. 2023 Nov 9;13:19520. doi: 10.1038/s41598-023-46740-6 (PMC10636012; doi:10.1038/s41598-023-46740-6)
Supplement: Supplementary file 1 — Supplementary Information. [file 41598_2023_46740_MOESM1_ESM.docx]

**Supplementary Information**

For the Figure 3C- RT-PCR analysis (showing both SM-A and SM-B expression in SCI bladders), the full-length gel is shown below.


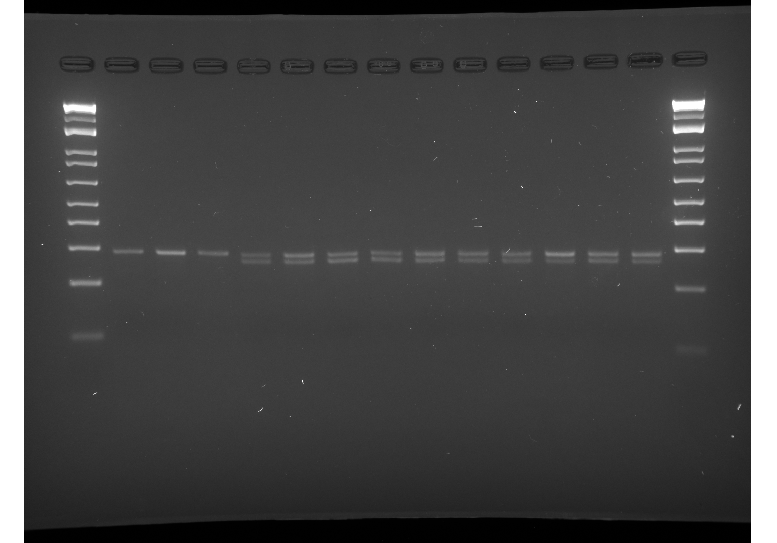


This gel was from a single experiment. The final gel in the image is cropped from the full-length gel. The full-length gel does NOT have any additional columns that are not reflected in the final image, except for one more ladder at the end. Original full-length gel image, at multiple exposures, are available upon request. Multiple gels were NOT stitched together for this final image.
